# Impact of Online Group Psychoeducation and Support Sessions on Receptivity Towards Digital Mental Health Care During the COVID-19 Pandemic: A Pilot Study

**DOI:** 10.1007/s41347-022-00281-3

**Published:** 2022-09-24

**Authors:** Cynthia M. Castro Sweet, Elizabeth J. Li, Sara Sagui-Henson, Camille E. Welcome Chamberlain, Myra Altman

**Affiliations:** 1 Clinical Research, Modern Health, San Francisco, CA USA; 2 Stanford Clinical Excellence Research Center, Stanford, CA USA

**Keywords:** Digital health, Mental health, Group processes, Health, Psychoeducation

## Abstract

While social distancing was crucial to slow the COVID-19 virus, it also contributed to social isolation and emotional strain. This pilot study evaluated the impact of stand-alone psychoeducational group sessions designed to build social connectedness and space for people to learn about mental health during the pandemic. The study examined if offering the stand-alone group sessions increased uptake of and receptivity to additional mental health services. People had access to free, online group psychoeducational sessions offered by a digital mental health platform company. Sessions were offered to (1) employees who had mental health benefits offered through their employer, and to (2) members of the general public. Session formats included discussions, didactic lectures, and workshops, were facilitated by a mental health provider, and used live video conference technology. Topics included race and identity, stress management, coping with political events, relationship issues, and self-compassion. First-time session registrations were tracked from June 2020 to July 2021 on 6723 participants (3717 benefits-eligible employees and 3006 from the general public). Among the employee subsample, 49.5% attended a group session as their first use of any available service on the platform; 52.5% of these employees sought additional services after their first session. In anonymous post-session surveys of employees and members of the general public, 86% of respondents endorsed knowledge increases, 79.5% reported improved understanding of their mental health, 80.3% endorsed gaining actionable steps to improve mental health, 76.5% said that they would consider group sessions in addition to therapy, and 43.5% said that they would consider group sessions instead of therapy. These results suggest that scalable, brief group psychoeducational sessions are a useful conduit to mental health care and have potential to reach people who may not otherwise access available mental health services.

## Introduction

When the COVID-19 pandemic began, loneliness and social isolation drastically increased as a result of the physical distancing methods introduced to slow the spread of the virus (Hwang et al., 2020; Roy et al., 2021; Rozenkrantz et al., 2020). The psychological toll of the COVID-19 pandemic was further compounded by incidents of racial discrimination, race-based violence, and political polarization that occurred during the height of pandemic lockdowns (Bruine de Bruin et al., 2020; Cheah et al., 2020; DeVylder et al., 2020; Dubey et al., 2020). The compounding interpersonal and social stressors of the pandemic led to a surge in mental health concerns on a global scale (Kumar & Nayar, 2021), and increased the demand for mental health care in an already resource-constrained environment (U.S. Department of Health and Human Services, Health Resources and Services Administration, National Center for Health Workforce Analysis, 2020).

Social connectedness and a sense of community are especially important for preserving mental health during times of heightened societal turmoil (Smith & Lim, 2020). The pandemic posed daunting challenges for connecting people with mental health services for support. Digitally delivered, remote-based mental health resources and support services rapidly emerged as a viable, scalable option to meet the surging demand for care during COVID-19 (O’Brien & McNicholas, 2020; Zayde et al., 2021). At the start of the pandemic, most one-on-one mental health care shifted to virtual meetings or phone calls, and many group therapy programs followed suit (Puspitasari et al., 2021). While digital delivery of mental health care grew considerably, less than half of the population with any mental illness received treatment in 2020 (Substance Abuse & Mental Health Services Administration, 2021), suggesting that barriers still existed that influenced people’s reluctance or inability to engage in care.

One recommended intervention to improve the uptake of mental health service utilization is group-based psychoeducation. This approach has been shown to be effective for increasing comfort in engaging with mental health services and reducing personal stigma regarding mental health challenges (Alvidrez et al., 2009; Taylor-Rodgers & Batterham, 2014). Group-based sessions where community members opt in to participate have been shown to be effective for engaging people in mental health care (Brown, 2018). Furthermore, single-session group psychoeducation sessions have been found to improve knowledge of mental health, reduce stigma around receiving treatment, and increase willingness to recommend seeking of mental health services (Lee-Tauler et al., 2018). Studies have found promising results of online, group psychoeducational sessions to improve mental health literacy and help-seeking attitudes and intentions (Tay et al., 2020; Taylor-Rodgers & Batterham, 2014), and improved mental health symptoms (Ali et al., 2021; Ghanbari et al., 2021; Sapru et al., 2018). However, these studies had small sample sizes, focused on specific mental health conditions, and/or consisted of a series of sessions. The purpose of this study was to examine the impact of stand-alone, online, group psychoeducation sessions on mental health service utilization, attitudes, and intentions. Specifically, we examined whether employees sought additional mental health services after their first experience of a group session, and whether all session participants reported improved attitudes and intentions for seeking mental health care after the sessions.

## Methods

### Design

This study was an observational, retrospective pilot study using real-world data of people who registered to attend online, group psychoeducational sessions offered by a digital mental health platform company (Modern Health, San Francisco, CA). We tracked available information from all people who registered for sessions from June 2020 to July of 2021 when data were extracted for analysis. The data used in analyses were originally designed for programmatic operations and not originally intended for research purposes. Available data included email addresses from registrations, platform utilization, and post-session survey ratings (described in more detail in the “Measurements” section). Available data fields were extracted from the company data warehouse through queries; data were then loaded into a dataset/data tables. Once unique cases were identified, the dataset was de-identified for analyses. The research was reviewed by WCG Institutional Review Board and was determined to be exempt from human subject research.

### Participants

Two distinct populations were included in the study: benefits-eligible employees and people from the general public. Criteria for all participants included in analyses were as follows: (a) registration for online, group psychoeducational sessions within the study time frame; (b) aged 18 years or older at the time of registration; (c) access to a smartphone or computer with Wi-Fi or cellular service in order to connect to the online sessions.

#### Benefits-Eligible Employees

These study participants could participate in the online group sessions because they received mental health care benefits from their employer. Employees had access to the digital mental health platform offered by the company, and that access included the online group sessions. Employees could register for the online group sessions either through a public webpage on the website of the mental health platform company, or they could establish a personal account on the platform and register through the platform portal. Unique email addresses were used to track employee registrations through either route.

#### Public Group Session Registrants

In addition to sessions provided to the benefits-eligible employees, the mental health company offered online group sessions free-to-the-public as a corporate social responsibility action during the COVID-19 pandemic. A public-facing web page described the sessions and had a registration link. The only information collected for the free-to-the-public sessions was the email address used for registration. Given the constraints on the data, these session registrants were included in limited analyses described in the “Data Analysis Plan” section. See Table 1 for a description of the data that were available on the benefits-eligible employees relative to the public group session registrants.

**Table 1:** Available data sources from benefits-eligible employees vs. participants from the community at large

**Available data**	
**Benefits-eligible employees**	**General public**
Session registration • Unique e-mail address • Session topics • Number of session registrations	Session registration • Unique e-mail address • Session topics • Number of session registrations
Demographic data • Gender (male, female, undisclosed) • Age in years	
Digital mental health platform utilization • Digital contents accessed • Number of coaching sessions • Number of therapy sessions	
Post-session surveys • Satisfaction • Attitudes about mental health • Help-seeking intentions	Post-session surveys • Satisfaction • Attitudes about mental health • Help-seeking intentions

#### Recruitment Methods

Benefits-eligible employees learned about the sessions through direct communications sent from their employer and/or the platform company, primarily through direct email marketing and social media posts. Public registrants learned about the existence of the free sessions through mass media communications sent from the mental health platform (i.e., postings on social media and the platform company website).

### Intervention

The online, group psychoeducational and support sessions (named “Circles”) are live community sessions delivered online. The sessions were initiated in response to the impact of social distancing restrictions imposed to slow the spread of the COVID-19 virus. The intent of the sessions was to create a forum for people to virtually connect with others and learn about and discuss their mental health during the COVID-19 pandemic. Sessions addressed a variety of topics such as follows: coping with uncertainty, race and identity, coping with racism, caregiving, parenting, improving sleep, improving health habits, stress management, coping with political events, relationship issues, self-compassion, building resilience, and combating negative thoughts. Session formats included live discussions, didactic lectures, and practical workshops. All sessions were facilitated by a mental health provider who was either a licensed clinician or certified professional coach in the network of providers managed by the mental health platform company. Individuals could register for an unlimited number of sessions, and all were offered at no charge to the attendee.

Sessions were designed to be stand-alone, meaning that they were designed to be self-contained, and not expected to be taken in a specific sequence. People could attend as many sessions as they wanted, and could repeat sessions that were offered more than once. Topics were repeated throughout the study time frame, and the dates/times and facilitators varied throughout the year. The variety of session topics, formats, dates, and facilitators was intentional in order to provide people options that were appealing and accessible to them. Session durations ranged from 60 to 90 min. As the sessions were launched during the height of COVID-19 pandemic social restrictions (i.e., June 2020), all sessions were conducted online using videoconferencing technology (either webinar format for large “listen & learn” didactic sessions, or video meeting format for workshops and discussions). The large didactic sessions could accommodate hundreds of participants and encouraged participants to ask questions through the chat feature. Workshops and discussions were intentionally smaller and could accommodate up to 50 participants; participants were encouraged to use their video cameras and audio connections to interact with the facilitator and other attendees during these sessions.

#### Digital Mental Health Services Platform

The online group sessions are a component of a comprehensive digital mental health platform. The full platform of services was only available to benefits-eligible employees and not to the general public attending the free-to-the-public sessions. The platform was available to employees through an app. As described in more detail in the “Measurements” section, we were able to separate the accounts of employees who accessed services on the platform before registering for their first group session from those employees who had no previous engagement on the platform before registering for their first group session.

Employees created an account on the platform with their email address and password of choice. The app surfaces questions that assess each individual’s clinical needs and care modality preferences to tailor care recommendations. Based on an individual’s baseline clinical severity and preference for care, recommendations are made to tailor services to meet their needs. Employees can follow the recommendations to connect to care, or can choose their own services if desired. In addition to the group sessions, options for care include the following: self-guided digital content (i.e., a resource library of self-help reading materials, audio files of meditation exercises); one-to-one telehealth sessions (video-delivered individual sessions) and in-app messaging with a licensed mental health specialist or certified professional coach; and the online, group psychoeducational sessions. Employees with full access to the platform could use any modalities of care and combine multiple modalities. We specifically focused on employees who utilized the online, group psychoeducational sessions as their first use of the platform.

### Measurements

#### Group Session Registration

An email address was the only element of data required to register for a group session. These addresses were the primary data source for all session registrations for both the benefits-eligible employees and for the general public accessing the free-to-the-public sessions. The first time a unique email address was used to register for a group session, an initial time stamp was created for that unique user. We then tracked the total number of subsequent session registrations with the same email address during the study period, along with the topics for each session.

#### Use of Digital Mental Health Service Platform

Additional data from the mental health benefits platform were available only for the benefits-eligible employees. These participants were identified when the unique email address that was used for a group session registration was also associated with a platform account. Platform service utilization and demographic data were extracted from these accounts. To examine engagement in other services on the platform, we tracked use of all available services on the platform relative to the date of the individual’s registration for their first group session, including (1) use of trackable, self-guided digital content (e.g., meditation exercises); (2) engagement in one-on-one sessions with either an in-network coach; or (3) therapist; and/or (4) registration for additional group sessions. Some elements of the digital content usage were not trackable with timestamps and thus were not accessible for analyses. Demographic data extracted from employee platform accounts included gender (male, female, or unknown) and age in years. No platform utilization data or demographic data were available for public registrants for the free-to-the-public sessions.

#### Post-Session Attitudes and Intentions

After each online group session, all attendees were asked to complete a survey of their impressions of the session. The surveys were requested from both benefits-eligible employees and from the general public that attended the free-to-the-public sessions. The surveys were anonymous using a single online survey link, and data could not be linked back to the mental health platform or registration records; therefore, we were unable to sort or separate the data based on whether the respondent was a benefits-eligible employee or a member of the general public. All session attendees were asked to rate the group session using the questions outlined in Table 2 using a Likert-type scale. These items were created to assess people’s satisfaction with the sessions and assess their intentions for taking additional action to care for their mental health. The questions were not designed for research purposes; the aim was to assess people’s impressions and intentions in a way that would not be overly burdensome.

**Table 2:** Post group-session survey questions

**Survey question**	**Response options**				
This Circle increased my understanding of my own mental health	Strongly disagree	Disagree	Neutral	Agree	Strongly agree
This Circle provided me with useful skills to improve my mental health	Strongly disagree	Disagree	Neutral	Agree	Strongly agree
This Circle provided me with actionable steps to improve my mental health	Strongly disagree	Disagree	Neutral	Agree	Strongly agree
How likely are you to use Circle sessions in addition to one-on-one therapy?	I am not sure	Not likely	Somewhat likely		Very likely
How likely are you to use Circle sessions instead of one-on-one therapy?	I am not sure	Not likely	Somewhat likely		Very likely

### Data Analysis Plan

#### Session Registrations

We calculated the total count of session registrations received during the study time frame, the total number of session registrations per unique email address, and separated these counts by registrations from benefits-eligible employees or from the community at large.

#### Use of Digital Mental Health Service Platform

To examine utilization on the mental health platform for the benefits-eligible employee participants, we calculated the proportion of members for whom the group session was their first activity of any kind on the platform. Among these participants, we calculated the percent that accessed additional mental health services or resources from the platform after their first group session, and the percent that completed coaching sessions, therapy sessions, or accessed additional digital content after their first group session. As we only had demographic data on the benefits-eligible employees, we calculated the percentages of participants identifying as female or male and calculated the average age of these participants.

#### Post-session Satisfaction, Attitudes, and Intentions

From the post-session survey data, we calculated the total number of surveys returned, a breakdown of the most frequent methods through which people learned about the sessions, and the percentage who endorsed the range of responses to the survey questions. As the surveys were collected anonymously, responses could not be linked to the mental health platform or registration records; therefore, all responses were combined for analyses and not separated based on whether the respondent was a benefits-eligible employee, or a member of the general public.

## Results

### Session Registrations

A total of 6723 unique emails were used to register for an online group session for the first time during the study time frame. Forty-five percent (45%, *n* = 3006) of these registrations came from the general public that had no access to the mental health services offered by the care platform. The other 55% of registrations (*n* = 3717) came from benefits-eligible employees who also had access to the full digital mental health platform and had a platform account with the identical email address that was used for group session registration. A total of 75 unique session topics were attended during the study time frame. Topics related to personal identity were most popular (e.g., “Healing Circles for Black Communities,” “Healing Circles for Asian Communities”), with 58% of all registrants (*n* = 3899) signing up for those group sessions.

Among all first-time group session participants (both benefits-eligible employees and members of the general public), 67% (*n* = 4504) registered for only one session; 17% (*n* = 1143) went on to register for a second session; 16% (*n* = 1076) went on to register for 3 or more additional sessions. The employees who had employer-sponsored access to the mental health platform were more likely to sign up for more than one group session compared to members of the general public, *X*^2^ (1, 6723) = 166.4, *p* < 0.001.

### Digital Mental Health Service Platform Utilization by Employees

Among the benefits-eligible employees, 45% (*n* = 1,673) identified as female, 14% (*n* = 520) identified as male, and the remaining 41% (*n* = 1,524) did not disclose gender identity. The average age of these participants was 36.9 years (range = 20 to 80 years).

Among the benefits-eligible employees who registered for their first group session during the study time frame (*n* = 3717), the group session was the first activity of any kind on the platform for 49.5% (*n* = 1841). These individuals did not create an account or engage in any other platform care activity until they signed up for their first online, group session. The other 50.5% (*n* = 1876) of the benefits-eligible employees who signed up for their first group session during the study period had previously set up an account and used at least one other care option before they attended their first online, group session.

Among the 1841 employees who had not previously used any platform services, 52.5% (*n* = 967) went on to use at least one additional care component of the mental health platform after their first group session. Specifically, 21.9% (*n* = 213) went on to utilize at least one telecoaching session, 9.3% (*n* = 90) utilized at least one tele-session with a licensed therapist, and 90% (*n* = 870) used some combination of digital content (i.e., self-guided reading material, recorded meditations and mindfulness exercises, and/or additional group sessions) after their first group session.

### Post-session Satisfaction, Attitudes, and Intentions

A total of 1639 feedback surveys were submitted after the group sessions. These surveys came from both benefits-eligible employees and from the general public registrants and could not be separated as they were collected anonymously (i.e., no identifiers were collected, and the database aggregated all survey responses and was not linked to the registration route).

When asked how they learned about the group sessions, 31% of survey respondents said that they learned about the sessions from an email sent from their employer, 18% learned from an email sent from the mental health platform company, 17% learned from a public website of the company, 13% learned from the mental health platform mobile app, and 10% learned from a friend or co-worker.

When asked about the impact of the group session, 79.5% of respondents agreed or strongly agreed that attending the session increased their understanding of mental health, 80.5% agreed or strongly agreed that the session gave them useful skills, and 80.3% agreed or strongly agreed that the session gave them actionable steps to improve their mental health. When asked about their receptivity towards using group sessions in addition to or instead of therapy, 76.5% said they were somewhat likely or very likely to use the group sessions in addition to therapy, and 43.6% were somewhat or very likely to use group sessions instead of therapy.

## Discussion

The results of this pilot study evaluation suggest that online, group psychoeducational sessions were attractive and acceptable to many people during the height of the COVID-19 pandemic, and reached a segment of the population that had not previously accessed mental health care through other modalities (i.e., through therapy, professional coaching, or digital content). Among the benefits-eligible employees who had full access to the platform mental health resources, over 1800 had not previously utilized their available services before their first group session; the online group session was their first point of entry. The finding that 52.5% of these benefits-eligible employees went on to use other elements of care after their first group session suggests that the sessions may have helped to increase interest in or receptivity to additional care among employees who had full access to mental health services but had not previously used them. Our results are consistent with the extant literature that single online group sessions can serve as an important starting point or conduit to additional care (Brown, 2018; Taylor-Rodgers & Batterham, 2014).

Less than one-third of the benefits-eligible employees who had not previously used their available services went on to use either therapy or coaching; the majority (90%) used digital resources and/or additional group sessions. This finding suggests that for many individuals, receiving care in a one-on-one setting from a coach or therapist is not necessarily the first step in attending to one’s mental health. The post-session survey responses about attitudes and intentions for additional mental health care add further support to this argument, with almost half (43.6%) of the respondents with survey responses (both employees and the general public) stating that they would be open to using group sessions *instead* of therapy, and the majority being open to using groups *in addition to* therapy. Individual visits with a mental health provider are not necessarily the right solution initially or exclusively for many people’s mental health needs.

Thousands of people from the general public also sought participation in these group psychoeducational sessions. Almost half of the registrations for the online group sessions were from people in the public who did not have access to the mental health platform through their employer. These people would not have received personal email invitations to participate, which implies that they found the group sessions through website searches, word of mouth, or from seeing content on the mental health company website or through company posts on social media. We found that benefits-eligible employees were more inclined to register for additional group sessions than the registrants from the general public; this may be because employees may have been made more aware of their employer-sponsored benefits as a result of the session, and took advantage of the available services afterwards. Given the limited data collected on the public session participants, we do not know if the registrants from the general public had access to mental health services elsewhere. While it is possible, it is also highly likely that access was quite challenging, both due to resource constraints imposed by the pandemic (World Health Organization, 2020), and by the pre-existing shortage of mental health services (U.S. Department of Health and Human Services, Health Resources and Services Administration, National Center for Health Workforce Analysis, 2020). The online and no-cost elements of the group sessions may have been particularly attractive and serve as an important public service to improve people’s exposure to and comfort with mental health care.

People were particularly drawn to the sessions that addressed topics of racial and ethnic identity, with over half of the registrants signing up for sessions around those topics. Given that the sessions took place between Fall of 2020 and late Spring of 2021, the popularity of these topics reflect the increased socio-political conflicts people were experiencing at the time, and the resulting impact these events had on people’s mental health and well-being, compounded by the stressors of COVID-19 (Cheah et al., 2020; Eichstaedt et al., 2021; Ibrahimi et al., 2020). Additional group sessions were continuously added as demand increased over time; therefore, the popularity of some session topics increased the likelihood that those sessions would be repeated more frequently, and thus continue to increase their prominence in the registration volume.

The sessions were intended to be psychoeducational and not group therapy, and the post-session survey ratings of the utility of the sessions suggest that they served their intended purpose. The majority of people endorsed positive gains on survey questions in terms of improving their understanding of their mental health and feeling more equipped with skills or actionable steps to work on their mental health. While we could not separate the post-session survey data on the benefits-eligible employees from the general public, and we were not able to determine engagement in additional mental health services from the general public, the combined positive sentiments of the post-session survey responses provide additional (though limited) evidence that online, single sessions of group psychoeducation can be helpful for improving help-seeking attitudes and intentions (Lee-Tauler et al., 2018).

### Limitations

While this study is a useful evaluation of real-world evidence of the impact of a digital mental health offering, the observational nature of the data poses challenges and limits the generalizability and interpretations of the findings. The study took place entirely during the COVID-19 pandemic when social isolation and stressors were prominent; while these conditions were a catalyst for the launch of the sessions, the social-political-environmental context of the time inevitably influenced what was observed during this study. With movement into a post-pandemic world, it will be important to routinely evaluate the group sessions and their impact on mental health service utilization, attitudes, and intentions to determine if the patterns seen in this study will endure as the larger environmental conditions change.

The different levels of data available on the benefits-eligible employees relative to the registrants from the general public constrained our ability to conduct group-specific analyses. It may be possible that the attitudes and intentions endorsed in the post-session surveys may differ by participant type (employee vs. general public), but we are not able to differentiate the results to examine this. The registration system for public users did not ask for any personal data beyond an email address, and limits our ability to understand more about the public participants (essentially, we only know the session topics of interest from registrations, and when those sessions were offered). Ideally, we would want comparable demographic information to examine differences/similarities in who sought out the group sessions, and confirmation of whether or not public participants had access to mental health services elsewhere. There is also a chance that people who had employer-sponsored access to the platform chose to register through the public registrations with different email addresses, and thus, we may have some false-negative cases of public registrations. However, this would result in an under-reporting of cases of people accessing services through the platform; we may be undercounting the people who utilize group sessions and no other modalities, despite having coverage for additional care.

These group sessions by design were quite diverse in terms of content topics, dates and times they were offered, formats, and facilitators that supported them. While this breadth of variety has its advantages, it also limits our ability to determine if there are any central, active ingredient(s) of these sessions that produce the desired effects. Additionally, while we were able to track registrations, we did not collect identifiers of session attendees; there is the likelihood that some individuals registered but did not attend. The observational nature of the real-world data also limits any implications about causality. Further studies will need more rigorous designs, control conditions, and intentional isolation of the hypothesized active ingredients to more precisely determine the cause(s) and their magnitude of effect.

The post-session surveys were optional, and there is the likelihood of a positive response bias in that those who benefitted the most from the sessions were more likely to complete the optional feedback survey. Additionally, while social connectedness, stigma reduction, and reduced loneliness/isolation are viable outcomes for group psychoeducational sessions, we did not have measurements of these constructs incorporated into the sessions. We have since added a post-session survey question inquiring if the session helped build a sense of community and are working on other ways to collect outcomes of interest on participants without adding burden to their experience. While the data had their limits and challenges, the group sessions have been highly popular and continue to endure. This provides us with future opportunities to improve upon data collection and tracking of individuals to more closely understand how the group sessions have helped guide people towards other elements of mental health care.

### Conclusions

The results of this evaluation suggest that scalable, brief, online group educational sessions can be a useful conduit to draw people to mental health care and can be an important element in expanding the reach of mental health services to people who may underutilize their available mental health resources. During the height of the COVID-19 pandemic when social distancing measures were widespread, the introduction of these online, group psychoeducational sessions were met with a large volume of interest, and attendees who shared their experiences revealed encouraging signs of their impact (e.g., encouraging people to take additional steps for their mental health). Online group sessions that are primarily psychoeducational and supportive in nature have exciting potential to be a point-of-entry; they can engage people who might not make full use of the mental health services available to them. Furthermore, some may choose to use them as a stand-alone or preferred option if given the choice. They can have a positive impact in improving people’s understanding about their mental health and giving them a sense of direction. They can also empower and encourage people to take further steps to access care for their mental health.
